# Mouse Phenome Database: an integrative database and analysis suite for curated empirical phenotype data from laboratory mice

**DOI:** 10.1093/nar/gkx1082

**Published:** 2017-11-10

**Authors:** Molly A Bogue, Stephen C Grubb, David O Walton, Vivek M Philip, Georgi Kolishovski, Tim Stearns, Matthew H Dunn, Daniel A Skelly, Beena Kadakkuzha, Gregg TeHennepe, Govindarajan Kunde-Ramamoorthy, Elissa J Chesler

**Affiliations:** The Jackson Laboratory, Bar Harbor, Maine 04609, USA

## Abstract

The Mouse Phenome Database (MPD; https://phenome.jax.org) is a widely used resource that provides access to primary experimental trait data, genotypic variation, protocols and analysis tools for mouse genetic studies. Data are contributed by investigators worldwide and represent a broad scope of phenotyping endpoints and disease-related traits in naïve mice and those exposed to drugs, environmental agents or other treatments. MPD houses individual animal data with detailed, searchable protocols, and makes these data available to other resources via API. MPD provides rigorous curation of experimental data and supporting documentation using relevant ontologies and controlled vocabularies. Most data in MPD are from inbreds and other reproducible strains such that the data are cumulative over time and across laboratories. The resource has been expanded to include the QTL Archive and other primary phenotype data from mapping crosses as well as advanced high-diversity mouse populations including the Collaborative Cross and Diversity Outbred mice. Furthermore, MPD provides a means of assessing replicability and reproducibility across experimental conditions and protocols, benchmarking assays in users’ own laboratories, identifying sensitized backgrounds for making new mouse models with genome editing technologies, analyzing trait co-inheritance, finding the common genetic basis for multiple traits and assessing sex differences and sex-by-genotype interactions.

## INTRODUCTION

The Mouse Phenome Database (MPD) is a collection of trait values and genotype data in the laboratory mouse. MPD differs from Mouse Genome Informatics (MGI) (1) in that quantitative phenotype data from individual animals are housed in MPD. These data are expertly curated by MPD staff and annotated with public ontology terms from Vertebrate Trait (VT) (2), Mammalian Phenotype (MP) (3), and Mouse Adult Anatomy (MA) (4) ontologies. An effort is underway to incorporate annotations to the Clinical Measurement Ontology (CMO) (5) and other relevant ontologies for integration with multi-species platforms such as GeneNetwork (6) and the Monarch Initiative (7). For each dataset, detailed protocol information and animal documentation (housing and environmental parameters) are collected and made available to MPD users and data consumers in standardized formats.

MPD houses phenotype and/or genotype data for over 1700 strains and populations. In order to provide the greatest amount of data aggregation in analysis, MPD accepts data from any verifiable mouse strain or population (correct nomenclature is required). Animals with associated data can be representatives of strains with defined genetic backgrounds or members of populations comprised of individuals with unique recombinant genomes. Strain types include inbred, recombinant inbred, F1 hybrid, transgenic, targeted mutant, chromosome substitution and Collaborative Cross. Populations include offspring from inbred strain F2s, backcross and other crosses found in the QTL Archive; Diversity Outbred mice (8); and UM-HET3 mice as used by the National Institute on Aging Interventions Testing Program (9). We provide an integrated and growing suite of visualization and analysis tools for phenotype data and provide a genotype retrieval interface to enable users to integrate and interpret genome-phenome relations across the database.

MPD facilitates a number of research applications, including the ability to select strains with the ideal combination of phenotypic characteristics for generating engineered mutations on virtually any genetic background (e.g., CRISPR/cas9 genome editing). MPD is also useful for:

- Accessing baseline and treatment data
- Providing validated protocols and relevant data collected under those protocols
- Choosing optimal strains for:
  - ∘ Modeling human diseases and conditions
  - ∘ Choosing sensitive genetic backgrounds for engineered mutations
  - ∘ Quantitative trait locus (QTL) analysis
  - ∘ Physiological studies
  - ∘ Drug studies
- Mining data in other ways:
  - ∘ Elucidating shared genetics for correlated traits
  - ∘ Discovering genotype-phenotype relationships
  - ∘ Formulating hypotheses and testing *in silico*
  - ∘ Studying sex differences and sex-by-genotype interactions
  - ∘ Assessing replicability

MPD has consistently provided rigorous curation of mouse experimental data to help alleviate issues associated with reproducibility and replicability (10,11). By structuring mouse phenotyping studies, annotating them to controlled vocabularies and developing integrative tools that rely on the unique value of these data, MPD facilitates access and reuse of primary phenotype data, enabling cross-species comparisons and ultimately assuring relevance to human studies.

Here, we present a new version of MPD that has been re-engineered using Web 2.0 technologies to facilitate interactive data exploration and quantitative analysis. We demonstrate several advances and features of this new design, including rigorous analyses implemented in the R statistical language, API capabilities and modernized interactive graphics.

## NEW FEATURES AND IMPROVEMENTS

Since our last NAR report, the entire MPD system has been updated and the web application has been redesigned. We have made the following major improvements to enhance user experience:

- R statistical computing language is employed to provide rigorous statistical analyses
- Data visualizations are upgraded and highly interactive
- Multiple, widely accepted ontologies are used for annotation
- ‘MyMPD’ provides stable user accounts for repeatable analysis on stored selections
- Retrieval of genotype data is streamlined toward integrative analysis
- Data access API is available for tool prototyping, offline analysis and use by other resources
- Look-and-feel is updated
- Search and navigation are improved
- Results tables are filterable, searchable, and sortable

### Search and navigation

Improved search capability provides the quickest way to find and select data of interest. Users can search to locate data for a strain, panel, procedure, treatment, keyword, investigator, contributing center, project, measurement, ontology term, gene, or a genomic region. An auto-suggest list is provided as users type in their search terms.

A set of links is available in the header of each page (not shown) where users can access a pull-down menu full of browsing options, as well as links to ‘About’ MPD and ‘MyMPD’, which, when in use, holds a customized collection of user-selected data (details below). In addition to navigating from the header of any page, tabs are available on the homepage (not shown), indicating what type of data users can access and how to find it (phenotype, genotype, strains, panels, QTL Archive, protocols), as well as links to a listing of data contributors and guidelines on how to submit data. Tabs are also available for quickly accessing available downloads and toolbox demos.

Three simple steps are iterated on the homepage for using MPD: (i) find and select data, (ii) go to MyMPD and (iii) analyze. A quick-start tutorial is available on the homepage to learn how to use MPD. New features and new datasets are listed near the bottom of the homepage with a link to view all entries where readers will find that many new datasets have been added to MPD since our last NAR report.

### Phenotype data

An MPD ‘project’ is composed of a submitted dataset, metadata (including annotations to ontology terms and controlled vocabularies), a protocol (workflow, equipment, reagents, procedural steps) and animal documentation (housing and environmental parameters). Projects are associated with investigators and primary publications of the data. A project contains data tables to which MPD’s summary, visualization and analysis tools, can be applied, as well as a table of strains tested and sample sizes, documented updates, and download options for access to primary data.

**Figure 1.**
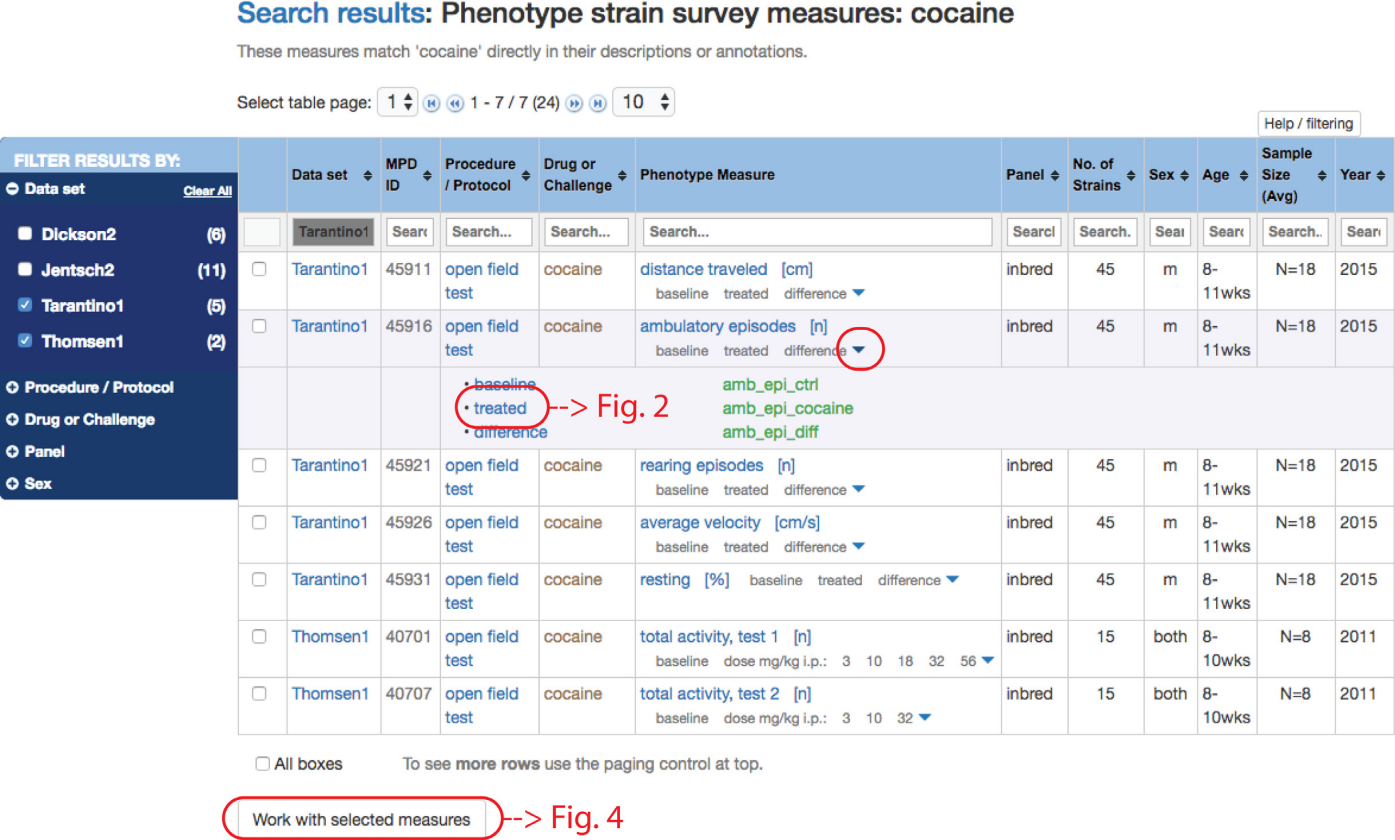
Search results tables are filterable by dataset, procedure, treatment, strain panel, or sex (see blue facet panel to the left); sortable (up/down arrowheads for each column); and searchable (search box for each column). A quick primer for getting the most out of these pivot tables is available through the link at the top of the table: ‘Help/filtering’. In this search result for ‘cocaine’ we have chosen datasets Tarantino1 and Thomsen1 in the facets panel (left). Measures in a set are indicated by the down arrowhead (red circle) in the ‘Phenotype Measure’ column. Clicking on the down arrowhead reveals a listing of measures that make up the set ‘ambulatory episodes’. Green text indicates variable names for single measures.

To illustrate the new phenotype sector, we use an example where the search term is ‘cocaine’. A search results table is shown in Figure 1. From the search results table, users can get directly to the project page where the associated publication is listed as well as additional information about participants, funding sources, and experimental design (not shown). The search results table also contains direct links to protocol information for each row in the table. In the ‘Phenotype Measure’ column, users will find rows of either single measures or measures in sets, e.g. repeated measures, which are indicated by down arrowheads. As illustrated in Figure 1, clicking on an arrowhead will expand the view, listing individual measures that make up the set.

**Figure 2.**
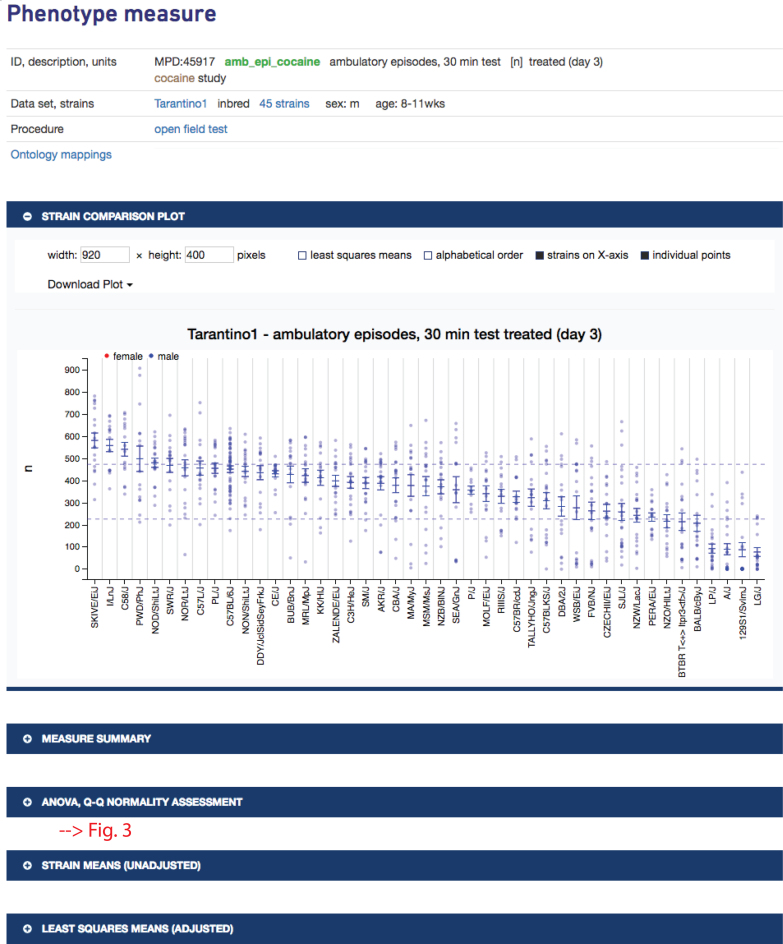
Phenotype measure information is at the top and includes measure ID, variable name, description, units, intervention (if applicable), project to which it is associated (link to project information and associated publication), population type, number of strains (link to table of strains tested with source links, if available), sex, age, method (direct link to protocol), and ontology mappings (clicking on link opens listing of annotated terms). Plot, summary statistics, ANOVA results, and Q–Q plot are available by expanding on the blue bars. Strain means and individual animal data points are shown in the plot. Dashed lines represent ±1 standard deviation, making it easy to identify outlier strains. Plotting options are shown above the plot along with download options. *Data are from project Tarantino1 (MPD:459)*.

**Figure 3.**
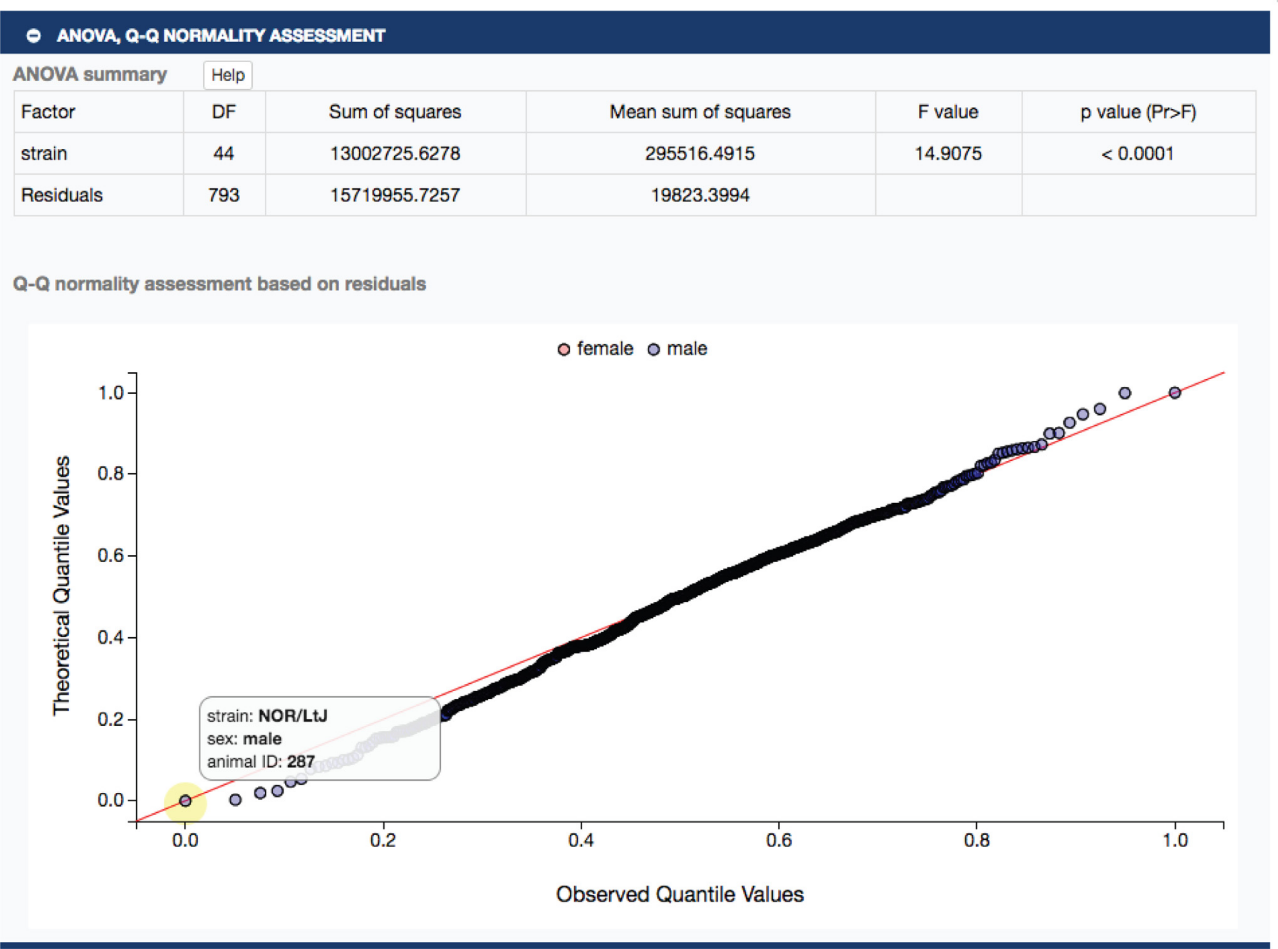
ANOVA results (upper panel) and Q–Q normality plots (lower panel) are available. Hovering (yellow highlight) over data points reveals strain, sex and animal identification number.

In Figure 1, clicking on ‘treated’ will take the user to a single measure plot as shown in Figure 2. The strains tested exhibit a wide range of phenotypic diversity for ambulatory episodes after cocaine treatment, as shown in the plot. Below the plot, a user will find expandable sections with summary statistics, ANOVA results and a Q–Q normality assessment, all implemented in R. For example, clicking on the latter expansion bar would open to a table of ANOVA results and a Q–Q plot as shown in Figure 3. These results indicate that there is a significant strain effect and that the data are normally distributed, providing a layer of quality assurance for users wanting to re-use these data.

For each row in the search results table, there is a checkbox to the left (see Figure 1). Users may select one or all measurements for direct analysis of the data or create a private collection of user-specified data (MyMPD). Once measures are selected, the user may click on ‘Work with selected measures’ to choose one of these options. A measure does not have to be in MyMPD to visualize and get summary statistics. MyMPD is particularly useful when unrelated measures across the database are of interest and there is a need to collect the data into a single set for analysis. Measures can be selected and placed in MyMPD folders which are created and managed by the user.

**Figure 4.**
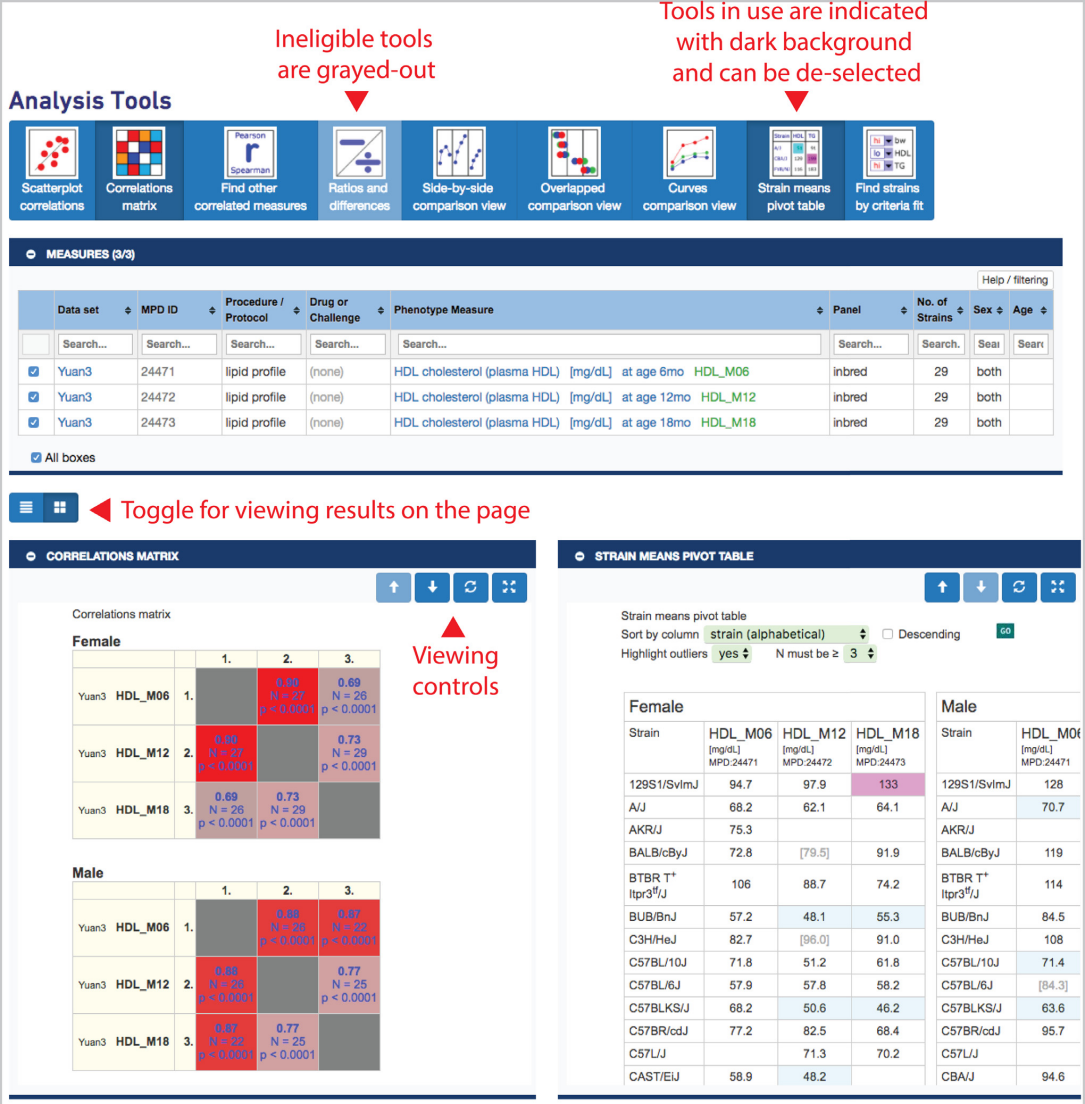
MPD toolbox provides a collection of tools for analysis on user selected measures. In this example the ‘Ratios and differences’ tool is ineligible (since three measures are selected) and the ‘Correlations matrix’ and ‘Strain means pivot table’ have been selected (darker background). Results are shown below, in this case, side-by-side. A toggle allows the user to view the results in a stacked manner as well. Each tool can be open or closed independently by clicking on the blue bar. Measures can be selected/deselected and results are automatically updated. *Data are from project Yuan3 (MPD:244)*.

Whether analyzing directly or analyzing through MyMPD, a user will end up on the MPD toolbox page as shown in Figure 4. A collapsible table opens to their selected measures, once again with checkboxes allowing the user ultimate flexibility in managing their collection such that subsets of measures and different combinations of measures can be chosen for analysis. As shown in Figure 4, as a new feature, eligible tools are in bright blue and ineligible tools are grayed-out, depending on the set of selected measures. As measures are selected and deselected, results are automatically updated. Additional modular tools for multivariate phenotype analysis and trait meta-analysis are being integrated in our tool set. We welcome collaborative contribution of additional robust modules.

### Genotype data

**Figure 5.**
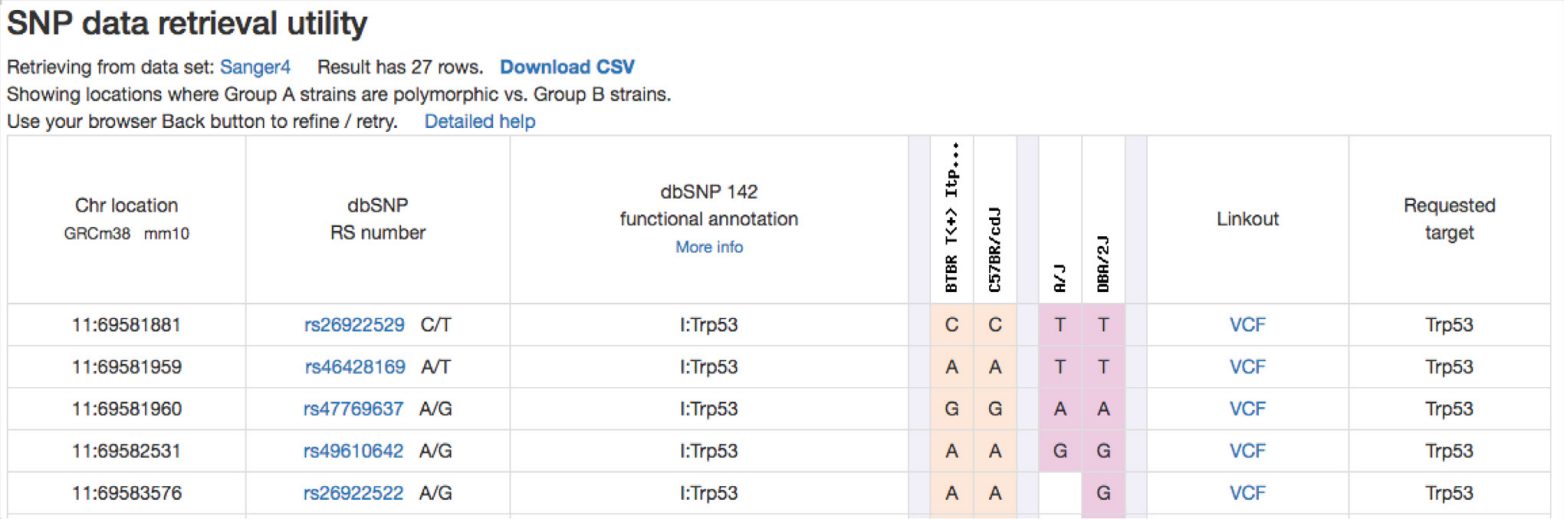
The MPD SNP tool allows comparison of multiple strain sets across several SNP data resources. Only the first five rows of this retrieval are shown. There are linkouts to NCBI dbSNP per rs number. The ‘VCF’ link retrieves data from the Sanger website for a particular location. Users can download SNP retrievals in .csv format.

We have incorporated new genotype datasets since our last NAR paper, including Sanger data for 37 inbred strains (80+ million genomic locations) and UNC mega-MUGA data for 77 Collaborative Cross strains and their founder strains (76+ thousand genomic locations). These datasets have been added to the existing collection of data from inbred, recombinant inbred, and chromosome substitution strains. Users can access the SNP-retrieval utility through the ‘Genotype’ tab on the homepage or through this quick link: https://phenome.jax.org/genotypes. We provide a SNP query form (single page) where users can enter gene symbols (MGI), coordinates (in bp or Mbp), or rs numbers either singly or in various combinations (up to 50 items may be listed). Users can select desired flanking sequence, and results can be filtered on dbSNP functional annotations. Users can query entire chromosomes or even the entire genome (these options are not currently available for the Sanger dataset due to its large size). Figure 5 shows a representative SNP retrieval table. This particular example illustrates the power of this tool where strain sets can be chosen based on phenotype data, and SNPs that reflect potential phenotypic differences can be readily identified. MPD staff will perform large, complex queries upon request (offline), making retrievals available as .csv files to MPD users.

### Strains and panels

**Figure 6.**
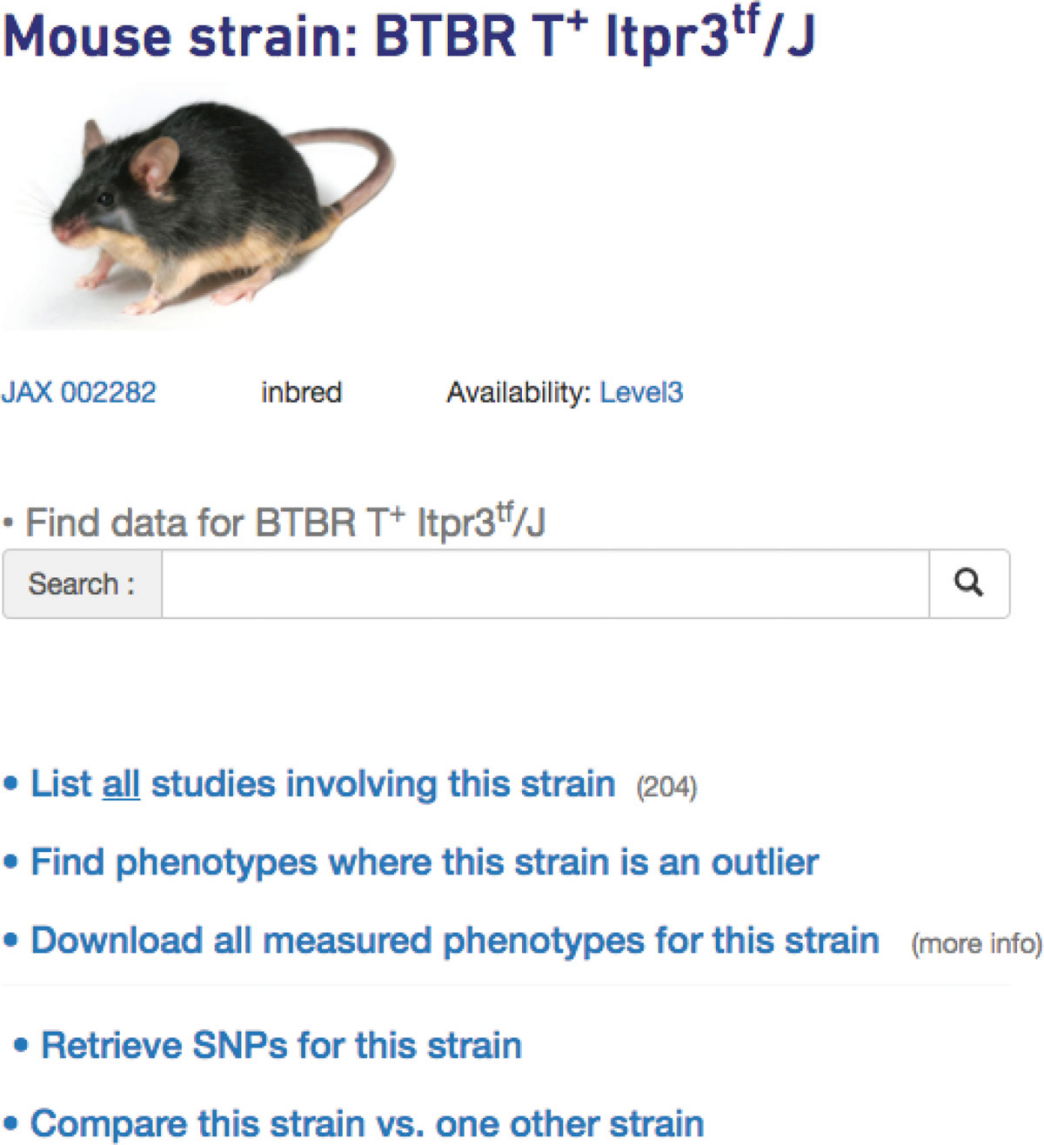
The strain hub page contains an image of a representative mouse, links to phenotype and genotype data, as well as provides a search box for data relevant to that strain. Users may also identify measures where their strain of interest is an outlier.

Phenotype and genotype data can be accessed through the ‘Strains’ or ‘Panels’ tab on the homepage (not shown). The user is taken to a listing of strains or panels—depending on which was chosen—where they can click on a strain or panel of interest. For example, clicking on the BTBR T^+^ Itpr3^tf^/J link on the strains page would take users to a hub page for this strain where links to all data are available, including phenotype and genotype data, as shown in Figure 6. The search box is prominent on this streamlined page, encouraging users to utilize our improved search capabilities (see above). From the strain hub page, users can also click on a link to ‘Find phenotypes where this strain is an outlier’. Results are filterable, searchable, and sortable. There is also an option to download all phenotypes for the strain of interest. Users can sort the resulting data by Z-score and use their criteria to identify the most extreme measures. From the strain hub page, users may also click on the link to ‘Compare this strain vs. one other strain’. After selecting a second strain, users are given the option to compare phenotype data or genotypes between the strains.

### QTL archive

The QTL Archive is a collection of quantitative trait locus (QTL) studies across populations and species and can be accessed through a tab on the homepage (not shown). Each study has phenotype and genotype data for members of a population derived from a specified cross. A list of phenotypes, an Excel archive file, and a .csv file formatted for use with R/qtl (12) are available. Most pages include original analysis scripts and other files as well as a list of associated MGI-curated genomic features. Users can locate a dataset of interest, download the data, and reproduce or extend analyses locally using software such as R/qtl, J/qtl (13), QTLRel (14), DOQTL (15) or the new R/QTL2 (http://kbroman.org/qtl2/).

### Interactive use of MPD data

MPD will serve as the primary point of phenotypic data curation for systems genetics resources such as GeneNetwork (6) and Diversity Outbred and Collaborative Cross projects (16). Benjamini and colleagues have developed a web application any researcher can use to evaluate the replicability of their phenotyping discovery (17), thereby improving the replicability of published studies and ensuring investments in research yield timely, cost effective and consistent results. This service has accessed MPD data to estimate the laboratory interactions and will be more closely integrated in the future. Further, phenotypic abnormalities have been accessed and integrated into analyses by the Monarch Initiative which aims to integrate, align, and re-distribute cross-species phenotype data (7). This will enable quantitative comparison of cross-species phenotypes, facilitating the identification of animal models of human disease through phenotypic similarity.

## GETTING AND SUBMITTING DATA

### Bulk and programmatic access

Bulk data downloads are available at https://phenome.jax.org/downloads in .csv format so that Excel can be used in local environments. A set of public API endpoints is available for programmatic access to specific phenotype data (individual animal data or strain means), metadata, and analytics results (all returned in JSON format). For more information, see https://phenome.jax.org/about/api.

### Data submission

Most of the data in MPD are voluntarily contributed by investigators worldwide. Projects can have one of many study designs. MPD collects data from baseline characterizations, diet studies, drug and alcohol studies, treatment vs. control studies, aging and longevity studies, challenge/pathogen studies, and more. We accept data from all strain types and populations. For phenotyping projects, we require a dataset as well as measurement descriptions and units. Submission of introductory content (succinct paragraph) is encouraged to help users quickly understand the study. In addition to the dataset and measurement descriptions, we require a detailed protocol and information about housing and testing environments. Research resource identification numbers (RRIDs) (18) are being included to unambiguously identify reagents and resources used in the study. Funding sources and other acknowledgements can be added to the project. If available, a primary publication and related publications can be linked to the project. Although not a requirement, we encourage investigators to publish their findings in peer-reviewed journals. Most MPD projects are associated with a primary publication, providing a layer of quality assurance for data consumers. From an investigator's perspective, in many cases submission to MPD satisfies journal mandates for public release of data.

We are currently implementing an interface for data contributors so that they may directly assist in curating their own data and creating data dictionaries to their specifications. This added feature will streamline the entire submission process, making data publicly available more rapidly than currently possible. Until this feature is released, data contributors should follow our data submission guidelines found at https://phenome.jax.org/about/contributedata, which will be updated once the new curation interface is operational. Submissions and questions should be sent to phenome@jax.org.

## IMPLEMENTATION AND PUBLIC ACCESS

The database (PostgreSQL) is organized into several schemas including: core (core functionality), snpdata (SNP genotype data sets), userdata (user collections), statout (analytics results), and limsdata (data captured from phenotyping LIMS systems). The web application and user experience were recently redesigned. There are now about 140 distinct page templates being utilized. We introduced some new user experience elements such as search suggestion typeahead and jQuery tablesorter for sortable filterable tables and interactive D3 visualizations.

We utilize two CentOS Linux virtual machines, one handling the web server and database, and the other handling computational work. The technology stack of our public-facing web application currently includes NetScaler, Apache, PostgreSQL database, Python, Flask, Bootstrap, JavaScript, jQuery modules such as tablesorter and D3.js for data visualizations. The stack for our computational server (not public-facing) includes Python for running functional tests and three docker containers each running Plumber, which gives access to the R-based analysis web service end points and provides load balancing (haproxy). Data communications between machines / resources is implemented using RESTful API endpoints and JSON.

The software development team uses Git and Bitbucket to manage code; JIRA for project management of our agile process, including tracking of releases, sprints, and issues; and Confluence for documentation. We use a two-week iteration cycle with public releases every six weeks. Automated testing is done at the unit and functional level, the latter being implemented in Groovy, using Geb and Spock. Unit testing is done with the Python built-in unittest module. Careful review by curation staff and data contributors is also an essential part of the validation and release process.

The statistics and analysis team develops analytic scripts in R. These scripts are hardened and installed on the computational server and connected to service API endpoints using Plumber. Currently these computational services are accessed both offline at data-accession time for long-running analyses and in real-time for quick analyses such as correlation of two traits.

## CITING MPD

For a general citation of the MPD resource, researchers should cite this article and use RRID:SCR_003212. The following citation format is suggested when referring to MPD datasets: Investigator(s) name(s) [last name, first initial, middle initial]. Title of project. MPD:project symbol [such as Smith1]. Mouse Phenome Database web resource (RRID:SCR_003212), The Jackson Laboratory, Bar Harbor, Maine USA. https://phenome.jax.org [Cited (date)].
